# Melanocortin receptor agonist NDP-α-MSH improves cognitive deficits and microgliosis but not amyloidosis in advanced stages of AD progression in 5XFAD and 3xTg mice

**DOI:** 10.3389/fimmu.2022.1082036

**Published:** 2023-01-10

**Authors:** Eleonora Daini, Eleonora Vandini, Martina Bodria, Wenjie Liao, Carlo Baraldi, Valentina Secco, Alessandra Ottani, Michele Zoli, Daniela Giuliani, Antonietta Vilella

**Affiliations:** ^1^ Department of Biomedical, Metabolic and Neural Sciences, Laboratory of Molecular and Cellular Neurobiology, University of Modena and Reggio Emilia, Modena, Italy; ^2^ Department of Biomedical, Metabolic and Neural Sciences, Pharmacology Unit, University of Modena and Reggio Emilia, Modena, Italy

**Keywords:** 5XFAD, 3xTg, cognitive deficits, neuroprotection, NDP-α-MSH

## Abstract

**Introduction:**

Alzheimer’s disease (AD) is the most frequent cause of dementia and still lacks effective therapy. Clinical signs of AD include low levels of endogenous melanocortins (MCs) and previous studies have shown that treatment with MC analogs induces neuroprotection in the early stages of AD.

**Methods:**

We investigated the neuroprotective role of MCs in two transgenic mouse models of severe AD using 5 and 7 month-old (mo) 5XFAD mice and 9 and 12 mo 3xTg mice. These mice were subjected to a chronic stimulation of MC receptors (MCRs) with MC analogue Nle4-D-Phe7-α-melanocyte stimulating hormone (NDP-α-MSH, 340 μg/kg, i.p.). Mouse behavior and ex-vivo histological and biochemical analyses were performed after 50 days of treatment.

**Results:**

Our analysis demonstrated an improvement in cognitive abilities of AD mice at late stage of AD progression. We also showed that these protective effects are associated with decreased levels of hyperphosphorylated Tau but not with Aβ burden, that was unaffected in the hippocampus and in the cortex of AD mice. In addition, an age-dependent NDP effect on glial reactivity was observed only in 3xTg mice whereas a global downregulation of p38 mitogen-activated protein kinase was selectively observed in 7 mo 5XFAD and 14 mo 3xTg mice.

**Conclusion:**

Our results suggest that MCR stimulation by NDP-α-MSH could represent a promising therapeutic strategy in managing cognitive decline also at late stage of AD, whereas the effects on neuroinflammation may be restricted to specific stages of AD progression.

## 1 Introduction

Alzheimer’s disease (AD) is the most frequent cause of dementia, affecting about the 70% of those 50 million people suffering from dementia world-wide. Moreover, its prevalence is expected to spread dramatically in the coming years, due to the global increase of population mean age. More specifically, AD prevalence is believed to double by 2050 and disease-related costs are estimated to rise from a trillion of dollars in 2018 to 2.5 trillions by 2030, delineating an urgent public health problem (1). Therefore, it is important to develop effective and safe therapies to relieve memory loss and the global cognitive decline in AD patients, thus reducing the burden of this disease (2). Currently, no treatments are available to halt or reverse AD. In fact, the only available drug therapies for AD approved by the Food and Drug Administration (FDA) are targeting symptoms, such as cholinesterase inhibitors (donepezil, rivastigmine and galantamine) and memantine, which is a N-methyl-D-aspartate (NMDA) receptor antagonist, and novel treatments have mostly failed in clinical studies, except for the expensive monoclonal antibody aducanumab (3) whose marketing authorization was however recently rejected by EMA (EMA/112932/2022 Rev.1); additionally, treatment of late stage AD with these drugs shows moderate results (4, 5).

Despite an intense research activity, clinically available drugs for AD that target disease-specific pathways are currently lacking: many promising treatments in phase II clinical trials have not passed to phase III due to lack of efficacy and/or adverse events (6). A possible explanation of the poor clinical benefit of the studied drugs may be the inability to reach the right target at the right stage of AD. Indeed, since β-amyloid (Aβ) may accumulate in the brain for more than 10 years before the first clinical manifestation of AD, it could be difficult to treat these patients, especially those with a longer disease history (6, 7). In this context, the development of disease-modifying agents with pleiotropic and or multitarget activities is urgently needed (7, 8). There is a large literature showing that Aβ complexes and plaques, as well as neurofibrillary deposition, impair neuronal survival leading to altered intracellular axonal transport, cellular shrinking, synaptic loss and apoptosis (7, 9). Moreover, the Aβ plaque accumulation leads to the recruitment of inflammatory cells favoring neuronal loss (10, 11). Further evidence demonstrates an important role of microglia, the resident central nervous system (CNS) macrophages, in Aβ pathology (12). In fact, in healthy brain microglia exert several crucial roles, by sensing and detecting signs of injury, undergoing morphological and biochemical changes leading to an activated phenotype; these processes could be triggered through different targets including melanocortin receptors (MCR). Synthetic melanocortin agonists have been previously reported as a promising therapeutic tool towards a wide range of central neurological syndromes (13–15). In particular, NDP-α-MSH interaction with melanocortin receptor 4 (MC4R) (16), the most abundant MCR in the CNS and the only one present in microglia and astrocytes, has been already shown as an effective treatment for ischemia and early and moderate AD in experimental mouse models (10, 17–22). However, no studies have explored yet the potential of MCR agonism in severe models of AD.

The aim of this study is to assess the effects of MCR agonist NDP-α-MSH in two transgenic mouse models of AD, 5XFAD and 3xTg mice, that markedly differ as regards the relationship between cognitive alterations and neuropathology. In fact, in 5XFAD mice the accumulation of intracellular Aβ (iAβ) is transient and very precocious (2-3 months of age, mo) and is followed by the deposition of extracellular Aβ (eAβ) and neuroinflammation that are associated with the appearance of cognitive decline (5-6 mo) (11). On the other hand, in 3xTg mice, cognitive decline (5-6 mo) precedes accumulation of iAβ and eAβ and neuroinflammation that become apparent at 9 (iAβ) or 14 (eAβ) mo, being indeed markedly less intense than in 5XFAD mice (23). Therefore, while both AD models lead to prominent cognitive impairments at comparable age, the development of neuropathology is much slower and less intense in 3xTg mice. For these reasons, in this study we analyzed the possible effects of chronic NDP-α-MSH treatment on cognitive and neuropathological alterations in 5 and 7 mo 5xFAD mice and in 9 and 14 mo 3xTg mice.

## 2 Materials and methods

### 2.1 Animals

In this study two animal models of AD were used. Seven and 12 mo 3xTg male mice, harboring three mutations associated with familial AD (APP_Swe_, MAPT_P301L_, and PS1_M146V_) on a congenic C57BL/6J background, and control littermates were purchased by The Jackson Laboratory (Stock number 008880) (10, 24). 5XFAD mice, which co-overexpress a triple-mutant human APP (Swedish mutation: K670N, M671L; Florida mutation: I716V; London mutation: V717I) and a double-mutant human PS1 (M146L and L286V mutations), and B6SJL mice were purchased from Jackson Laboratories and bred at Centro Stabulario Interdipartimentale, Unimore. Experimental 3 and 5 mo 5XFAD mice were obtained by crossing hemizygous 5XFAD mice with B6SJL breeders. All mice were kept in conditioned rooms with stable temperature (21°C) and humidity (60%), on a light/dark cycle of 12 hours (h). Food and water were available ad libitum and body weight was recorded throughout the entire observation period. All animal procedures were approved by the Committee on Animal Health and Care of the University of Modena and Reggio Emilia and Italian Ministry of Health (protocol number: n°974/2016-PR) and conducted in accordance with National Institutes of Health guidelines.

### 2.2 Drugs and procedures

[Nle4-D-Phe7]-α-Melanocyte Stimulating Hormone trifluoroacetate salt (NDP-α-MSH, here on simply referred as NDP) (kindly provided by Professor Paolo Grieco from the University of Naples Federico II) is a synthetic melanocortin analogue with long-lasting biological activity and acts as an MCR 1-5 agonist (except MC2R) and reach the CNS after systemic injection (17, 21, 25). Different cohorts of mice received a daily intraperitoneal (i.p) injection of NDP or saline (sal, vehicle) for 50 days at the dose of 340 μg/kg at the following experimental ages: 3 or 5 mo 5XFAD mice, and 7 or 12 mo 3xTg mice. NDP dose was chosen on the basis of our previous reports (10, 21, 26).

### 2.3 Behavioural analysis

On day 45 of treatment, mice were subjected to Morris Water Maze (MWM) procedure to assess spatial learning and memory as previously described (21, 27). Briefly, animals were placed in a circular white pool with a diameter of 120 cm filled with water at 23± 2°C made opaque by adding white non-toxic paint and allowed to swim for 60 seconds (sec) or until they found the location of a hidden circular platform with 11 cm diameter (escape latency). During this *learning phase* mice were trained with four consecutive trials per day (starting from a different quadrant with a different visual cue at each trial) for four days and escape latency was recorded and averaged across the daily trial session. To assess memory retention, the platform was removed on day 5 and the animals were allowed to swim for 60 sec (p*robe test*), and the latency to reach the platform area, total entries in the platform area and the % of time spent in target quadrant were evaluated.

Behavioural tests were performed by an operator unaware of the treatment to avoid bias and were conducted in a sound-proof room. All data are shown as mean ± standard error of the mean (SEM) and were analysed by one-way analysis of variance (ANOVA), or two-way repeated measures ANOVA followed by the Bonferroni’s test, using the statistical package SPSS (version 26). Differences were considered significant with p-value (p) < 0.05 *; p < 0.01 **; p < 0.001 ***; p < 0.0001 ****.

### 2.4 Sample preparation and histology

Ninety minutes (min) after behavioral test (day 50^th^ of the study), 5 and 7 mo 5XFAD and 9 and 14 mo 3xTg mice were anesthetized with isoflurane and sacrificed for histological analysis according to established protocols. Briefly, intracardial perfusion was performed with cold 4% paraformaldehyde (70 mL/7 min) preceded by an infusion of 50 ml of saline containing heparin sodium (5000U/L); organs as brain, liver, spleen and kidneys were dissected out. The brains were postfixed in the same solution for 12 h, rinsed in a 30% sucrose-PBS solution for two days. The brains were then frozen using dry ice, and coronal 40 μm thick sections series were cut at a cryostat, washed three times in cold PBS and stored at -20°C in a glycerol-PBS solution until use.

### 2.5 Immunohistochemistry of brain sections

Brain slices were processed according to established protocols to test AD alterations; the following antibodies (Abs) were used: mouse anti-human Aβ/APP 6E10 (epitope human Aβ1-16, 1:500, Signet, #9300-10), rabbit anti-ionized calcium-binding adapter molecule 1 (IBA1, 1:1000, Wako, #019-19741) and rabbit anti-glial fibrillary acidic protein (GFAP, 1:2000, Dako, #Z0334).

A pre-treatment with formic acid was performed for anti-Aβ 6E10. Vectastain ABC-HRP (#PK-4002; #PK-4001) kit was used for peroxidase diaminobenzidine stainings (see **Table 1**); slices were placed on gelatinized glass slides, dehydrated and mounted with Eukitt^®^ mounting medium.

**Table 1:** List of primary and secondary antibodies used for immunohistochemistry (IHC) and western blot (WB).

	Marker or antibody	Host	Reactivity	Working dilution	Catalogue code	Producer
IHC	6E10	mouse	human	1:500	9300-10	Signet
	IBA1	rabbit	human/mouse	1:1000	019-19741	Wako
	GFAP	rabbit	human/mouse	1:2000	Z0334	Dako
	ABC-HRP anti-rabbit	goat	–	1:200	PK-4001	Vector
	ABC-HRP anti-mouse	horse	–	1:200	PK-4002	Vector
WB	ß-Tubulin	rabbit	human/mouse	1:3000	2146S	Cell Sign
	phospho-APP T668	rabbit	human/mouse	1:2000	3823S	Cell Sign
	phospho-Tau S202	rabbit	human/mouse	1:1000	39357S	Cell Sign
	phospho-Tau T181	rabbit	human/mouse	1:1000	12885S	Cell Sign
	p38 MAPK	rabbit	human/mouse	1:500	4511S	Cell Sign
	pErk	rabbit	human/mouse	1:1000	9101L	Cell Sign
	TNFα	rabbit	human/mouse	1:1000	3707S	Cell Sign
	Aß1-42	rabbit	human/mouse	1:1000	8243S	Cell Sign
	BACE-1	rabbit	human/mouse	1:1000	5606S	Cell Sign
	MC4R	rabbit	human/mouse	1:1000	ab75506	Abcam
	phospho-Tau S396	mouse	mouse	1:1000	9632S	Cell Sign
	phospho-JNK T183/Y185	rabbit	human/mouse	1:1000	9251S	Cell Sign
	anti-rabbit IgG HRP-linked	goat	–	1:3000	7074S	Cell Sign
	anti-mouse IgG HRP-linked	goat	–	1:3000	7076S	Cell Sign

### 2.6 Image and statistical analyses

Immunolabeled brain slices from wild type (Wt) and AD mice at different ages (5 and 7 mo for 5XFAD mice and 9 and 14 mo for 3xTg mice) were photographed using a Nikon Eclipse CiL microscope (10x objective) through a Nikon DS-Fi3 camera under constant light conditions. To avoid any bias, mice were encoded by numbers and analyzed by an operator unaware of the treatment or the genotype.

For 6E10 and IBA1 analysis, at least three images/mouse from cortex (CTX), hippocampal dentate gyrus (DG), cornu Ammonis (CA) 1, CA3 [Bregma between -1.70 and -2.06 mm from (28)] and dorsal subiculum (SUB) [Bregma between -2.92 and -3.40 mm from (28)] were converted to 32 bit grayscale and binarized applying a different threshold (shift from the background mean grey value) that was specific for each stain/area/mouse age (**Table 2**). Then, % of positive area, i.e., the area covered by pixels with gray value darker than the selected threshold, was automatically recorded through ImageJ software. Additionally, 6E10 positive plaques and IBA1 positive microglial cells were manually counted in a 500,000 µm^2^ area.

**Table 2:** Specific image settings used for the quantification of Ab-immunoreactivity.

		5XFAD		3xTg	
		5 mo	7 mo	9 mo	14 mo
CTX	6E10	40	65	10	10
	IBA1	20	10	20	15
	GFAP	30	25	nd	nd
DG	6E10	25	40	nd	nd
	IBA1	20	15	15	20
	GFAP	40	20	30	35
CA1	6E10	15	20	15	10
	IBA1	25	15	15	15
	GFAP	30	30	25	40
CA3	6E10	25	20	10	10
	IBA1	25	20	20	15
	GFAP	35	30	20	40
SUB	6E10	nd	nd	15	20
	IBA1	nd	nd	20	20
	GFAP	nd	nd	20	40

Thresholds used for 6E10, IBA1 and GFAP analyses in CTX, DG, CA1, CA3 and SUB of 5 and 7 mo 5XFAD mice and 9 and 14 mo 3xTg mice. Thresholds are expressed as grayscale units subtracted from the peak of mean grey value distribution. nd, not determined; CTX, cortex; DG, dentate gyrus; CA3, cornu Ammonis 3; CA1, cornu Ammonis 1; SUB, subiculum.

For GFAP immunostaining, the % of positive area was analyzed in 4 sections/area/mouse in hippocampal CA1, CA3, DG [Bregma between -1.70 and -2.06 mm) and SUB [Bregma between -2.92 and -3.40 mm from (28)] applying different thresholds through ImageJ (**Table 2**).

Data are expressed as mean ± SEM and were analysed by means of one-way ANOVA followed by Bonferroni correction. Statistical analyses were performed through SPSS software and p < 0.05 was considered for a significant difference.

### 2.7 Protein extraction and western blotting analysis

After 50 days of treatment, a separate group of 14 mo 3xTg (n=10) and 7 mo 5XFAD (n=27) mice and relative control littermates (C57BL/6J n=4 and B6SJL n=15, respectively) was sacrificed under deep anaesthesia through intracardiac injection of cold PBS. The hippocampus (Hip) was dissected and homogenized by mechanical disruption in Lysis Buffer (25 mM Tris/HCL, pH 7.4, 1.0 mM EGTA, 1.0 mM EDTA, 0.5 mM phenyl methylsulfonyl fluoride, aprotinin, leupeptin, pepstatin A (10 μg/ml each) and 100 mM Na_3_VO_4_). Cytoplasmic proteins were extracted and quantified by using standard protocol Coomassie^®^ reagent (Thermo Fisher Scientific, Waltham, MA USA) as previously described (29, 30). After quantification, proteins (30 µg for each sample) were denatured, electrophoretically separated, and transferred onto nitrocellulose membranes. The blots were then blocked and incubated overnight at 4°C with blocking buffer containing the primary Abs reported in **Table 2** (10, 21, 29, 31, 32). The following day, the membranes were incubated with a specific secondary anti-rabbit or mouse IgG-HRP-linked Ab for 1 h at room temperature. The membranes were analysed by the enhanced chemiluminescence system according to the manufacturer’s protocol (Millipore). The protein signals were quantified by scanning densitometry using a bio-image analysis system (Bio-Profil, Celbio, Italy) and expressed as relative optical density (OD) normalized over ß-tubulin (ß-Tub).

All data are shown as mean ± SEM. Values were analyzed by one way ANOVA followed by the Bonferroni *post-hoc*, using Graphpad Prism. Differences were considered significant with p value <0.05.

## 3 Results

### 3.1 Behavioral test

The hippocampus-dependent spatial learning and memory were assessed by the MWM task 45 days after sal or NDP treatment. The escape latency to find the position of the hidden platform in MWM during four consecutive days was considered as a learning index.

**Figure 1:**
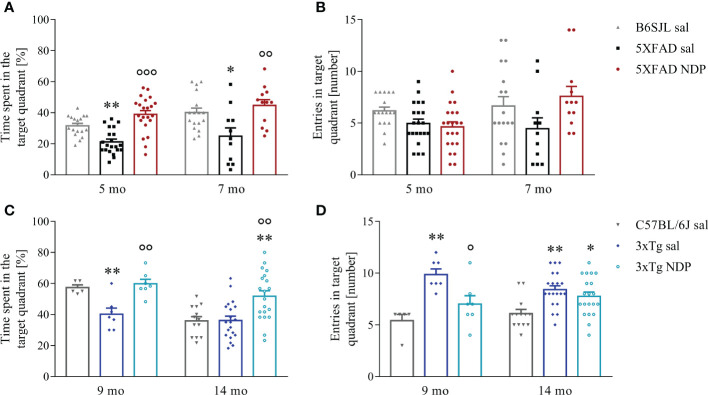
NDP treatment improves memory in AD mice. A decreased preference for platform searching in the trained target quadrant during the probe test in both 5XFAD **(A)** and 3xTg **(C)** is improved by NDP treatment at both experimental ages, indicating that in both AD mouse models the acquisition of the memory for the platform location is impaired; in addition, contrary to 3xTg mice **(D)**, in 5XFAD mice the number of entries in the target quadrant did not significantly change at any experimental age **(B)**. Both parameters were measured in the target quadrant during 60 sec of probe test; data are shown as mean ± SEM. and analyzed by means of one-way ANOVA followed by Bonferroni correction (* vs Wt; ° vs Tg sal; *,°p < 0.05, **, °°p < 0.01, °°°p < 0.001). Experimental groups: B6SJL sal (grey bar): 5 mo: n=17; 7 mo: n=17; 5XFAD sal (black bar): 5 mo: n=21; 7 mo: n=11; 5XFAD NDP (red bar): 5 mo: n=22; 7 mo: n=12; C57BL/6J sal (grey bar): 9 mo: n=6; 14 mo: n=13; 3xTg sal (blue bar): 9 mo: n=7; 14 mo n=20; 3xTg NDP (turquoise bar): 9 mo: n=7; 14 mo: n=20.

As concerns 5XFAD mice, the performance in a cue-guided training was not different between B6SJL and 5XFAD mice demonstrating that spatial learning abilities are preserved in both 5 and 7 mo experimental mice (daily escape latency, one-way repeated measures ANOVA; 5 mo: days of training F(1,57)=88.493, p<0.001, treatment F(2,57)=0.362, p=0.698; 7 mo: days of training F(1,37)=0.202, p=0.655, treatment F(2,37)=0.671, p=0.517; not shown). Twenty-four hours after the last training session, mice underwent a 60 sec probe test to measure the task performance regardless of the absolute/precise location of the hidden platform; the analysis of the performance was performed by measuring the % time spent in the target quadrant and demonstrated a significant decrease in 5XFAD mice that was counteracted by NDP treatment both at 5 and 7 mo even if the number of entries in the target quadrant was unchanged (one-way ANOVA, % time: 5 mo: F(2,29)=10.929, p<0.001; Tg sal vs Tg NDP p<0.001; 7 mo: F(2,39)=6.373, p=0.004; Tg sal vs Tg NDP p=0.005; number of entries: 5 mo: F(2,29)=0.854, p=0.437; 7mo: F(2,29)=2.355, p=0.109) (**Figures 1A, B**).

Similar results were also obtained with 3xTg mice; in fact, even if the capability to learn the position of a hidden platform in a cue-guided training was preserved in 9 mo and 14 mo 3xTg mice compared to C57BL/6J mice (daily escape latency, one-way repeated measure ANOVA; 9 mo: days of training F(1,16)=1.650, p=0.2617, treatment F(2,16)=0.188, p=0.830; 14 mo: days of training F(1,50)=14.654, p<0.001, treatment F(2,50)=0.819, p=0.447; not shown), the performance in the probe test was significantly impaired in 3xTg mice suggesting a spatial memory deficit. Of note, the treatment with NDP significantly improved mouse behavior (one-way ANOVA, % time: 9 mo: F(2,18)=11.343, p<0.001; Tg sal vs Tg NDP p=0.001; 14 mo: F(2,52)=8.918, p<0.001; Tg sal vs Tg NDP p=0.001; number of entries: 9 mo: F(2,18)=10.382, p=0.001; Tg sal vs Tg NDP p=0.021; 14 mo: F(2,52)=7.258, p=0.002; Tg sal vs Tg NDP p=0.720) (**Figures 1C, D**).

### 3.2 Immunohistological studies

#### 3.2.1 Amyloid plaques

**Figure 2:**
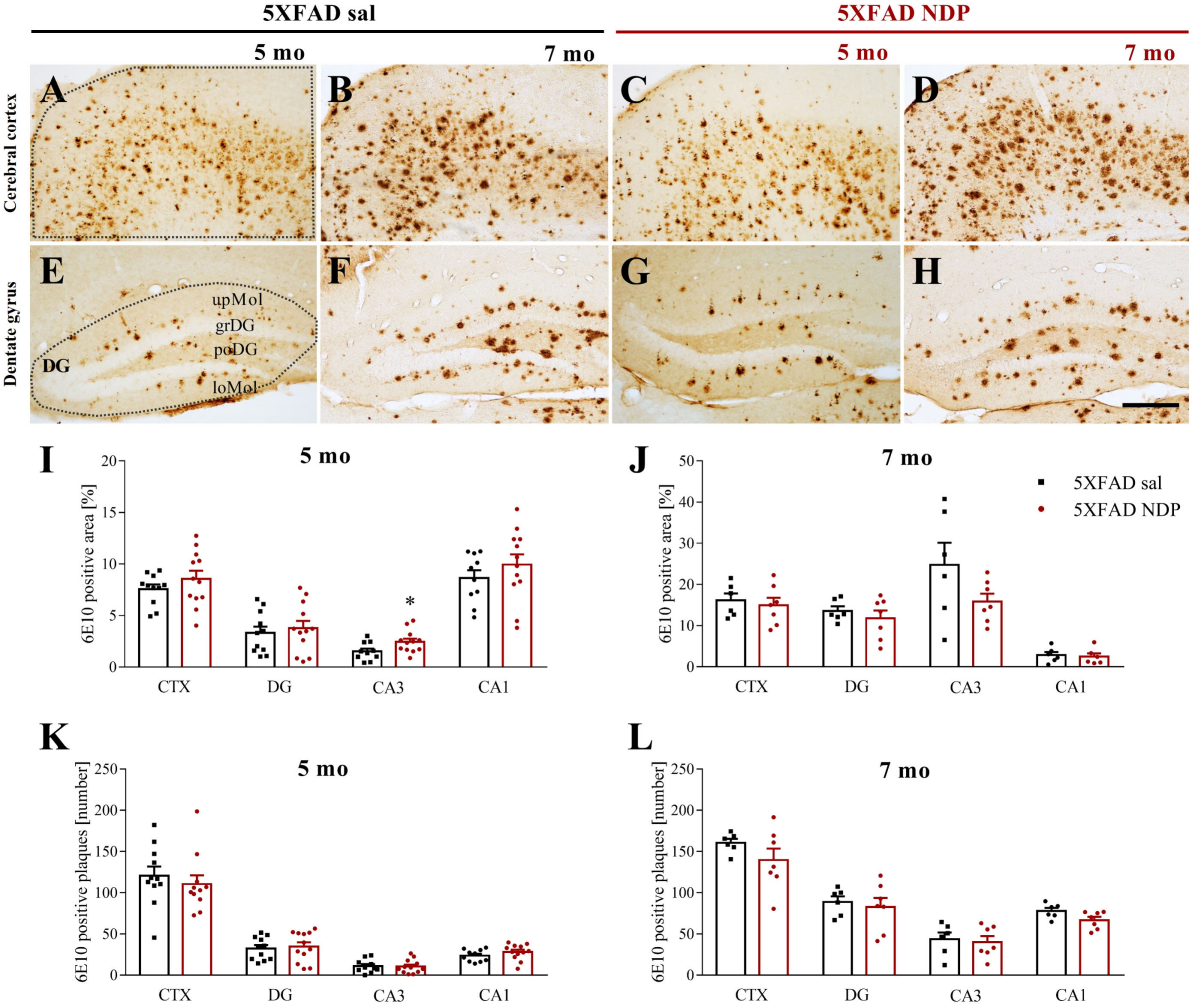
Amyloid deposition in 5XFAD mice is not influenced by NDP treatment at any stage of AD progression. Panels **(A–H)** Representative images showing 6E10+ plaque in CTX **(A–D)** and DG **(E–H)** of 5 and 7 mo 5XFAD treated with sal or NDP. Scale bar=200 µm. Panels **(I–L)** Analysis of 6E10+ plaque area and number in CTX, DG, CA3 and CA1 subfield of Hip of 5 mo and 7 mo of 5XFAD mice treated with sal (black bar) or NDP (red bar). Data are shown as mean ± SEM and were analyzed according to one-way ANOVA; *p < 0.05. Experimental groups: 5XFAD sal: 5 mo: n=10-11; 7 mo: n=6; 5XFAD NDP: 5 mo: n=12; 7 mo: n=6-7. CTX, cortex; DG, dentate gyrus; CA3, cornu Ammonis 3; CA1, cornu Ammonis 1; upMol, upper molecular layer; grDG, granular layer of DG; poDG, polymorph layer of DG; loMol, lower molecular layer.

**Figure 3:**
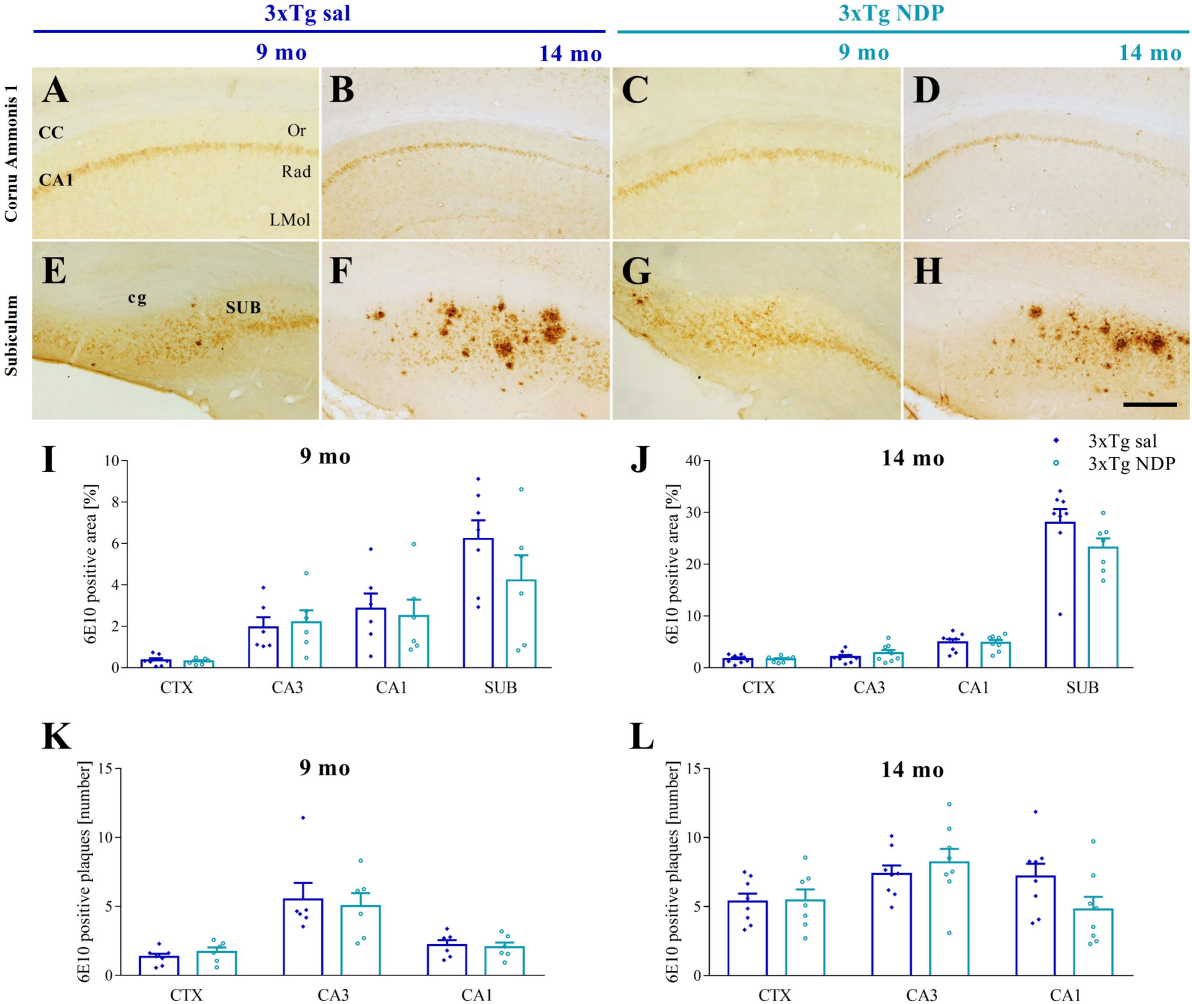
Amyloid deposition in 3xTg is not influenced by NDP treatment at any stage of AD progression. Panels **(A–H)** Representative images showing 6E10+ plaque in CA1 **(A–D)** and SUB **(E–H)** of 9 and 14 mo 3xTg mice treated with sal or NDP. Scale bar=200 µm. Panels **(I–L)** Analysis of 6E10+ plaque area and number in CTX, CA3 and CA1 subfield of Hip of 9 mo and 14 mo of 5XFAD mice treated with sal (blue bar) or NDP (turquoise bar). Data are shown as mean ± SEM and were analyzed according to one-way ANOVA. Experimental groups: 3xTg sal: 9 mo: n=6-7; 14 mo n=8; 3xTg NDP: 9 mo: n=6; 14 mo: n=7-8. CTX, cortex; CC, corpus callosum; cg, cingulum; CA3, cornu Ammonis 3; CA1, cornu Ammonis 1; Or, oriens layer; Rad, Radiatum layer; LMol, lacunosum moleculare layer.

The density of amyloid plaques and the mean % area occupied by Aβ immunoreactivity (ir) were measured in the CTX and in the Hip of brain slices stained with anti-6E10 Ab that detect both Aβ and APP. **Figure 2** provides representative images of hippocampal 6E10+ amyloid plaques and their relative quantification in 5 mo and 7 mo 5XFAD (**Figures 2A–H**) and 9 mo and 14 mo 3xTg mice (**Figures 3A–H**).

First, our analysis demonstrated the selective presence of Aβ^+^ protein aggregates in both AD models but never in respective B6SJL and C57BL/6J Wt mice (not shown). In 5XFAD mice, we did not find any significant effect of NDP treatment except for CA3 subfield of Hip. Specifically, at 5 mo the area covered by 6E10 ir in CA3 was increased (one-way ANOVA, F(1,21)=4.854, p=0.039) without any change in the plaque number (p=0.89) (**Figure 2**).

In the same way, in 3xTg mice we did not demonstrate any significant effects of NDP in analyzed parameters, suggesting that the amyloidosis process is not influenced by NDP at any stage of AD progression (for statistical report see **Supplementary Table 1**) (**Figure 3**).

#### 3.2.2 Glial fibrillary acidic protein staining

We used the GFAP marker to assess if astrogliosis, that increases in AD mice as a consequence of AD pathology, was modulated by NDP treatment.

**Figure 4:**
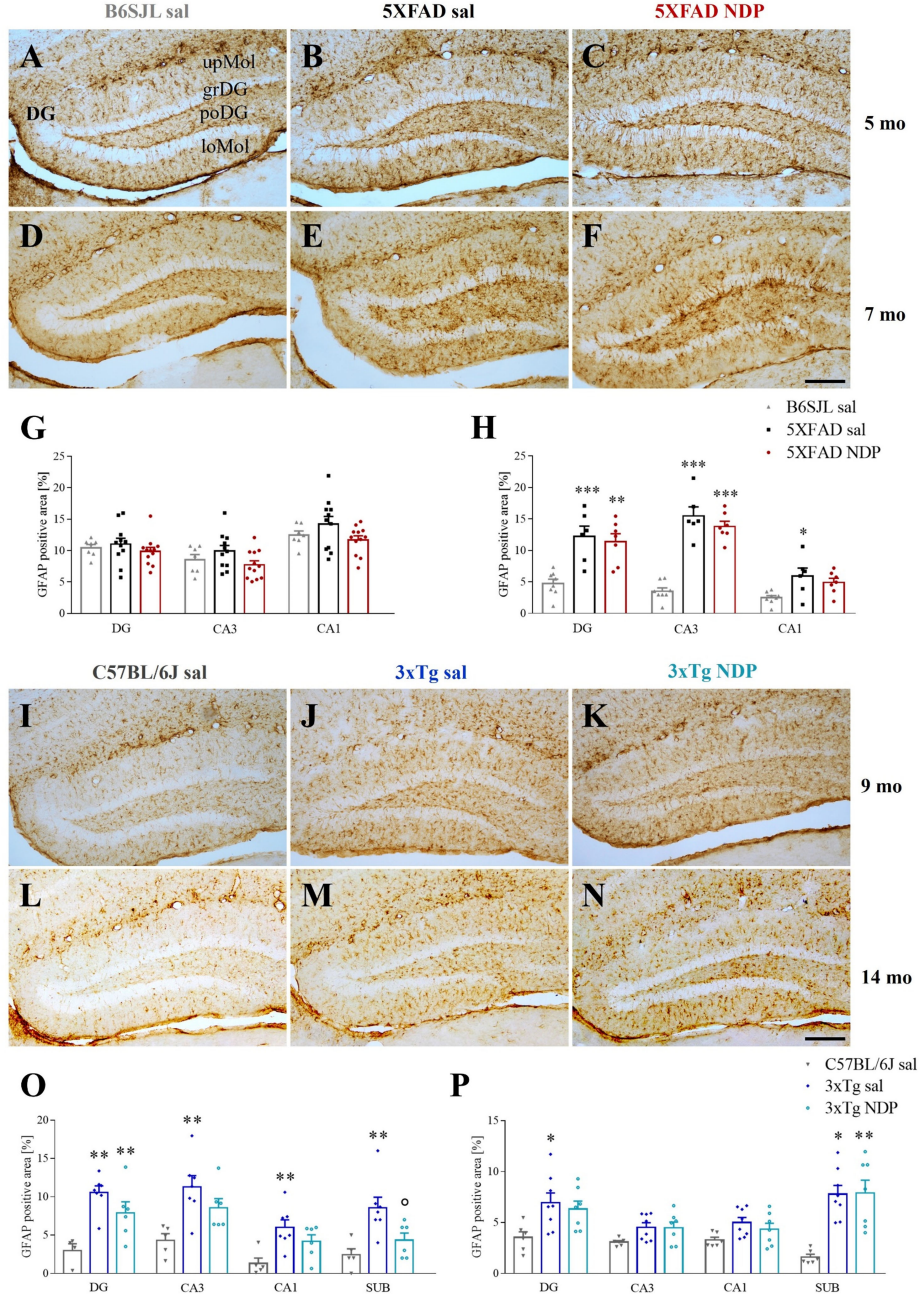
NDP treatment does not reduce astrocyte reactivity in the Hip of 5XFAD and 3xTg mice at different experimental ages. Panels A-F; I-N) Representative images showing GFAP ir in DG of 5 **(A–C)** and 7 **(D–F)** mo B6SJL and 5XFAD and 9 **(I–K)** and 14 **(L–N)** mo C57BL/6J and 3xTg mice treated with sal or NDP. Scale bar=200 µm. Panels **(G, H)** Analysis of the percentage of area coved by GFAP ir in DG, CA3 and CA1 of Hip of B6SJL (light grey bar) and 5XFAD treated with sal (black bar) or NDP (red bar). **(O, P)** Percentage of area coved by GFAP ir in DG, CA3 and CA1 and SUB of Hip of C57BL/6J (grey bar) and 3xTg treated with saline (blue bar) or NDP (turquoise bar). Data are shown as mean ± SEM and were analyzed according to one-way ANOVA followed by Bonferroni correction (* vs Wt; ° vs Tg sal; *,°p < 0.05, **p < 0.01, ***p < 0.001). Experimental groups: B6SJL sal: 5mo: n=7; 7 mo: n=8; 5XFAD sal: 5 mo: n=11; 7 mo: n=6; 5XFAD NDP: 5 mo: n=12; 7 mo: n=6-7; C57BL/6J sal: 9 mo: n=4-5; 14 mo: n=6; 3xTg sal: 9 mo: n=7; 14 mo: n=8; 3xTg NDP: 9 mo: n=6; 14 mo: n=7. DG, dentate gyrus; CA3, cornu Ammonis 3; CA1, cornu Ammonis 1; SUB, subiculum; upMol, upper molecular layer; grDG, granular layer of DG; poDG, polymorph layer of DG; loMol, lower molecular layer.

In 5XFAD mice, a marked astrogliosis was observed at 7 mo compared to Wt B6SJL mice without any effect of NDP treatment (for statistical report see **Supplementary Table 1**). On the contrary, in 3xTg mice a significant effect of NDP treatment was observed only in the SUB of 9 mo Tg mice (one-way ANOVA, F(2,17)=7.554, p=0.005; Tg NDP vs Tg sal: p=0.049) but not at 14 mo of age (**Figure 4**).

#### 3.2.3 Ionized calcium-binding adapter molecule 1 staining

The ir for IBA1, a marker of microglial cells, was analyzed to assess if increased microgliosis, that normally occurs in these AD mice, is modulated by NDP treatment.

**Figure 5:**
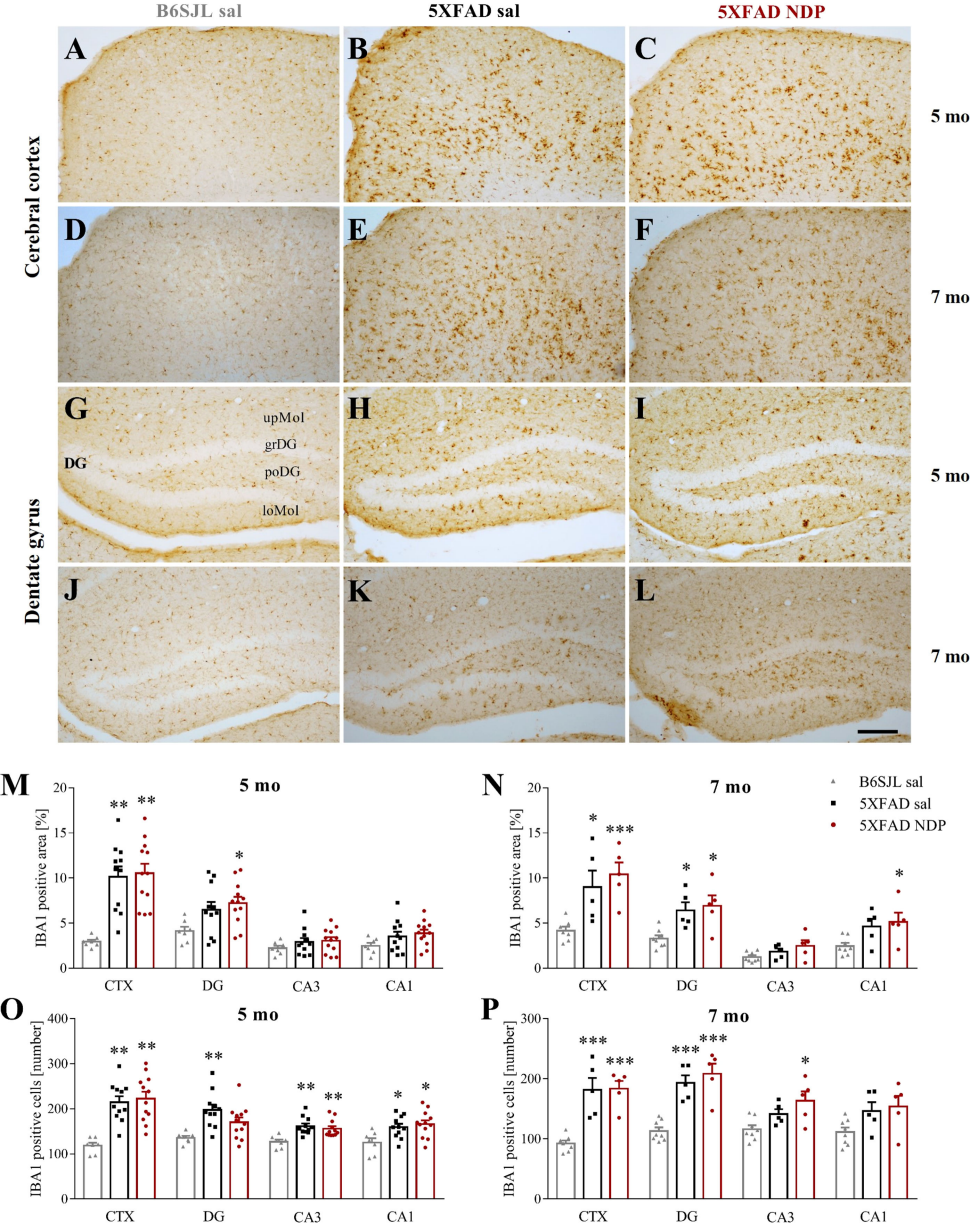
Microglial reactivity in 5XFAD mice is not influenced by NDP treatment at both early and late stage of AD progression. Panels **(A–L)** Representative images showing IBA1 ir in CTX **(A–F)** and DG **(G–L)** of 5 and 7 mo B6SJL control mice treated with saline and 5XFAD mice treated with saline or NDP. Scale bar=200 µm. Panels M-P) Analysis related to the percentage of area coved by IBA1 ir **(M, N)** and the number of IBA1+ cells **(O, P)** in CTX, DG, CA3 and CA1 subfields of Hip of 5 mo and 7 mo B6SJL (light grey bar) and 5XFAD mice treated with saline (black bar) or NDP (red bar). Data are shown as mean ± SEM and were analyzed according to one way ANOVA followed by Bonferroni correction (* vs Wt; *p < 0.05, **p < 0.01, ***p < 0.001). Experimental groups: B6SJL sal: 5 mo: n=7; 7 mo=7-8; 5XFAD sal: 5 mo: n=11; 7 mo: n=5; 5XFAD NDP: 5 mo: n=12; 7 mo: n=5. CTX, cortex; DG, dentate gyrus; CA3, cornu Ammonis 3; CA1, cornu Ammonis 1; upMol, upper molecular layer; grDG, granular layer of DG; poDG, polymorph layer of DG; loMol, lower molecular layer.

In 5XFAD mice, we found a significant increase in the number and/or in the area occupied by IBA1 ir in the CTX and in the DG, CA3 and CA1 of hippocampal formation without any effect of NDP treatment at both experimental ages (one-way ANOVA followed by Bonferroni correction, for statistical report see **Supplementary Table 1** and relative figure legend) (**Figure 5**).

**Figure 6:**
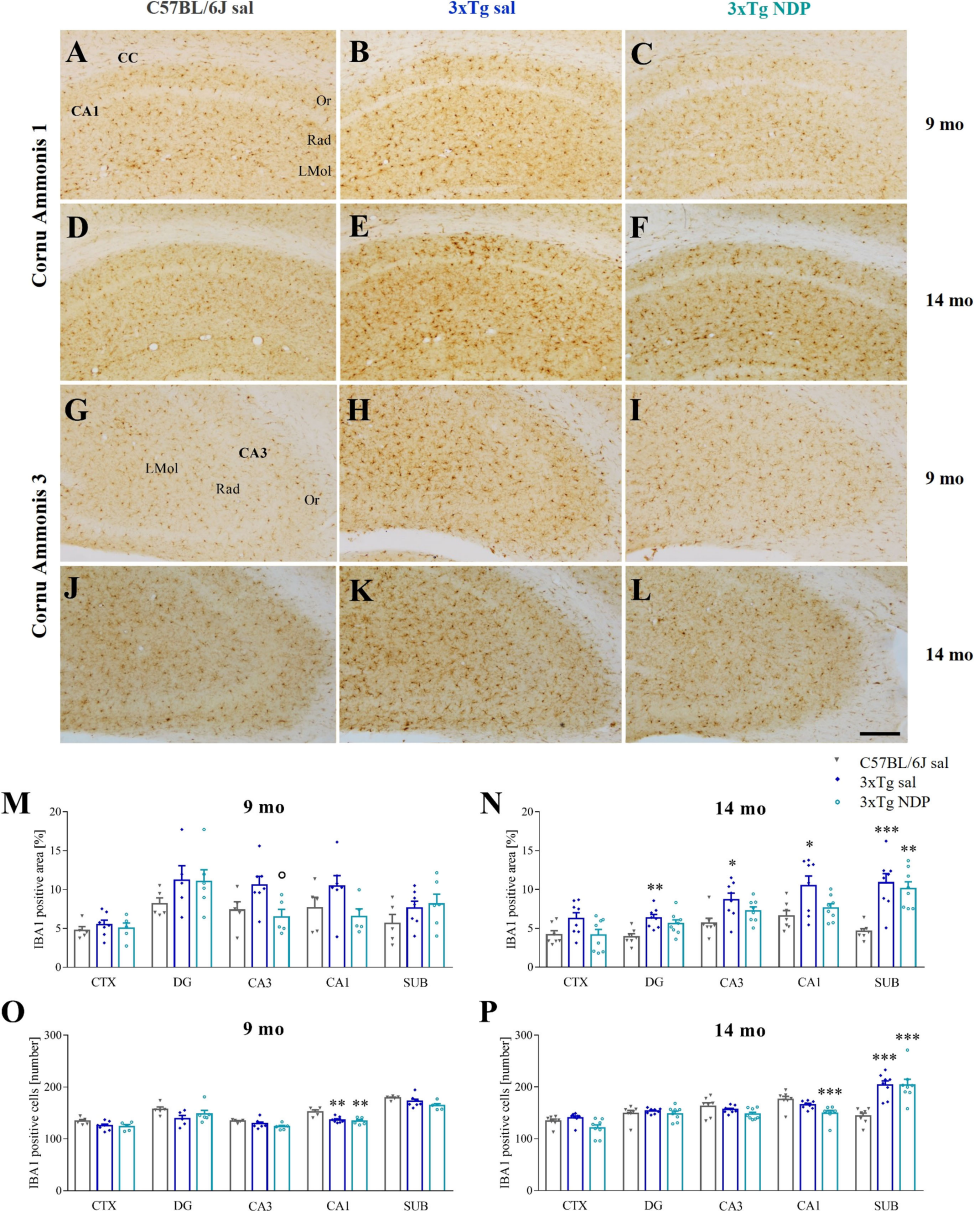
NDP treatment reduces microglial reactivity in the CA3 of 3xTg mice at early AD stages. Panels **(A–L)** Representative images showing IBA1 ir in CA1 **(A–F)** and CA3 **(G–L)** of 9 and 14 mo C57BL/6J (grey bar) and 3xTg mice treated with sal (blue bar) or NDP (turquoise bar). Scale bar=200 µm. Panels M-P) Analysis of the percentage of area coved by IBA1 ir **(M, N)** and the number of IBA1+ cells **(O, P)** in CTX, DG, CA3, CA1 and SUB subfields of Hip of 9 mo and 14 mo C57BL/6J (grey bar) and 3xTg mice treated with sal (blue bar) or NDP (turquoise bar). Data are shown as mean ± SEM and were analyzed according to one-way ANOVA followed by Bonferroni correction (* vs Wt; ° vs Tg sal *,°p<0.05, **p<0.01, ***p < 0.001). Experimental groups: C57BL/6J sal: 9 mo: n=5; 14 mo: n=7; 3xTg sal: 9 mo: n=5-7; 14 mo: n=8; 3xTg NDP: 9 mo: n=5; 14 mo: n=8. CTX, cortex; CC, corpus callosum; DG, dentate gyrus; CA3, cornu Ammonis 3; CA1, cornu Ammonis 1; SUB, subiculum; Or, oriens layer; Rad, Radiatum layer; LMol, lacunosum moleculare layer.

Compared to Wt C57BL/6J mice, in 9 and 14 mo 3xTg mice we observed slight changes in microglial reactivity, measured as the percentage of area covered by microglial cells and their absolute number, in several brain regions, i.e. CTX, several hippocampal subfields and SUB. Of note, the increased microgliosis was significantly counteracted by NDP treatment only in CA3 field of the Hip (one-way ANOVA, F(2,16)=4.263, p=0.036; Tg NDP vs Tg sal: p=0.049) at earlier stages of AD (for statistical report see **Supplementary Table 1** and relative figure legend) (**Figure 6**).

### 3.3 Biomolecular analysis

**Figure 7:**
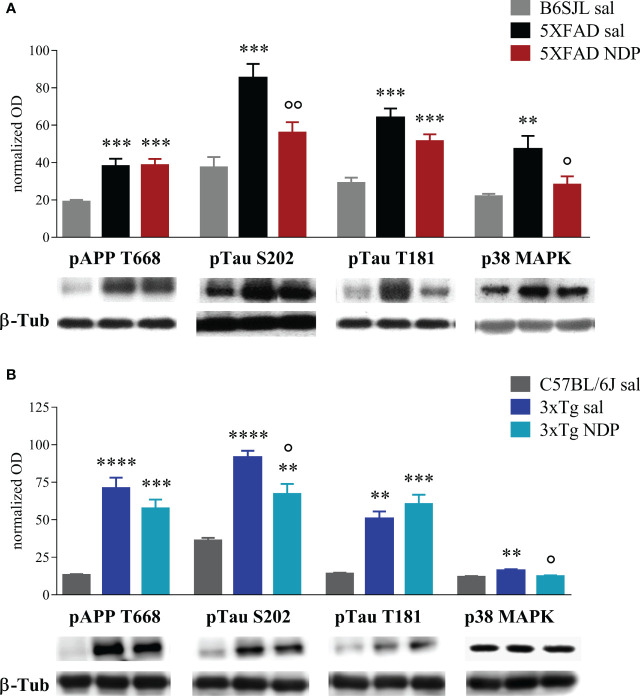
NDP treatment counteracts the expression of AD-related markers in the Hip of 7 mo 5XFAD and 14 mo 3xTg mice. **(A)** Protein levels measured by western blot analysis in B6SJL (light grey bar) and 5XFAD mice treated with sal (black bar) or NDP (red bar) and **(B)** in C57BL/6J (grey bar) and 3xTg mice treated with sal (blue bar) or NDP (turquoise bar). Ab-specific optical density (OD) was normalized over ß-Tub signal. Under the graphs representative immunoblots show hippocampal protein levels of ß-Tub, pAPP T688, pTau S202, pTau T181 and p38 MAPK. Data are shown as mean ± SEM and were analyzed according to one-way ANOVA followed by Bonferroni correction (* vs Wt; ° vs Tg sal °p < 0.05, **,°°p < 0.01, ***p < 0.001, ****p < 0.0001). Experimental groups: B6SJL sal: n=10-15; 5XFAD sal: n=8-12; 5XFAD NDP: n=10-15; C57BL/6J sal: n=4; 3xTg 14 mo: n=5; 3xTg NDP: 14 mo: n=5.

In 7 mo 5XFAD mice a significant increase in pAPP T668, pTau S202, pTau T181, p38 mitogen-activated protein kinase (p38 MAPK) (**Figure 7A**), and Aβ 1-42 and pTau S396 (**Supplementary Figure 2**) was observed with respect to Wt B6SJL mice; change in pTau T181 expression was not significantly altered by NDP treatment (Tg NDP vs Tg sal, p=0.096) while pTau S396 (F(2,25)=4.395, p=0.024), pTau S202 (F(2,27)=14.593, p<0.001) and p38 MAPK (F(2,25)=3.448, p=0.049) levels were significantly reduced (Tg NDP vs Tg sal, p=0.030, p=0.009 and p=0.025 respectively, for statistical report see **Supplementary Table 1** and relative figure legend).

In the Hip of 14 mo 3xTg mice, we detected a significant increase in the expression of pAPP T668, pTau T181 (**Figure 7B**) and BACE 1 (**Supplementary Figure 2**), with respect to Wt C57BL/6J mice; pTau S202 (F(2,11)=27.31, p<0.0001) and p38 MAPK (F(2,11)=9.661, p=0.0038) were significantly reduced in 3xTg mice by NDP treatment (Tg NDP vs Tg sal, p=0.014 and p=0.01 respectively, for statistical report see **Supplementary Table 1** and relative figure legend). However, the expression of pErk and TNFα were not significantly influenced neither by APP expression or by NDP treatment (see **Supplementary Figure 2** and **Supplementary Table 2**).

### 3.4 Toxicity

No signs of toxicity were recorded during the treatment with NDP (e.g. impaired spontaneous locomotor exploration and grooming, matted coat, diarrhea, lethargy, aggressiveness, hypothermia) and body mass of experimental 5XFAD and 3xTg mice is not influenced by NDP treatment and body weight changes were similar in all experimental groups (see **Supplementary Figure 1**). This is in agreement with our other works with NDP (10, 17, 18, 21, 22) and with recent studies that explained that these receptors, also known to be involved in an anorectic action, when hyperstimulated by chronic (or subchronic) treatments can mediate inverse agonist actions on energy control (16, 33).

## 4 Discussion

In this study we demonstrated that the NDP-mediated chronic stimulation of MCRs improves cognitive abilities of AD transgenic mouse models at late stage of AD progression. We also showed that these protective effects are associated with decreased hyperphosphorylated tau but not with Aβ burden, that was unaffected in the Hip and in the CTX of AD mice. In addition, an age-dependent NDP effect on microglial reactivity was observed only in 9 mo 3xTg mice whereas a global downregulation of p38MAPK was selectively observed in 7 mo 5XFAD and 14 mo 3xTg mice.

Specifically, hippocampus-dependent disrupted cognitive function and worsened performance of spatial memory task in 5 mo and 7 mo 5XFAD mice (**Figures 1A, B**) and in 9 mo and 14 mo 3xTg mice (**Figures 1C, D**) were significantly improved when NDP was daily administered for 45 days before behavioral testing. These results are in accordance with previous evidence obtained in other pre-clinical models at earlier stage of AD progression; in fact, we and others have demonstrated that stimulation of the hippocampal MC4R circuit relieves synaptic plasticity impairment in a mouse model of early-stage AD (10, 29, 34). We speculate that these changes could be mediated by the stimulation of MC3R and MC4R, two MCRs widespread in almost all cerebral areas including several key areas involved in memory such as the Hip (35). It is well known that the melanocortins are involved in a several physiological functions, including pigmentation (MC1), steroidogenesis (MC2), energy homeostasis (MC3), regulation both food intake and sexual function (MC4), neurogenesis, analgesia and inflammation (MC4), exocrine secretion (MC5) (36). Our present and past results demonstrate that NDP administered i.p. improves peripheral pathologies (hemorrhagic shock, sepsis, myocardial ischemia) and central ones (cerebral ischemia, head trauma and AD) and for the latter it is plausible to hypothesize the involvement of the MC3 and MC4 receptors as they are mainly expressed centrally, but it cannot be excluded an indirect involvement also of the peripheral melanocortin receptors. Other studies demonstrated that the treatment with other MCR agonists, such as α-MSH, restores fear memory impairment induced by IL-1β injection in dorsal Hip (34, 37). Globally, these observations indicate that the stimulation of melanocortin pathways reestablishes memory function even in advanced stages of AD.

To assess if improved behavior is associated with reduced neuropathology, we investigated the expression of AD-linked alterations at both histological and protein levels by means of immunohistochemical and western blot analyses, respectively. First, we measured the 6E10+ ir in cortico-hippocampal regions, the most affected by Aβ deposition in both 5XFAD and 3xTg mice; in addition, since 6E10 Ab recognises APP, iAβ and eAβ (11), the number of 6E10+ plaques as well as the area coved by 6E10 ir were considered. We did not find any significant effect in CTX and hippocampal subregions of either 5XFAD or 3xTg mice at both experimental ages, apart from an isolated increase in 6E10+ plaques in CA3 of 5XFAD mice at 5 months of age; of note, these observations were also supported by *ex vivo* analyses of relative pAPP T668 expression levels in the Hip of Wt and AD mice (**Figures 7A, B**). Differently from Aβ levels, our western blot analysis demonstrated that NDP treatment significantly reduced the level of hyperphosphorylated tau, specifically pTau S202 and pTau T181, in the Hip of AD mice. Taken together, these data suggest that NDP treatment and MCR stimulation in late phase of AD progression is not able to decrease the Aβ burden in severe mouse models of AD, such as 5XFAD and 3xTg mice, but is able to reduce tau phosphorylation that is known to better correlate with cognitive abilities (38).

Emerging evidence implicates MCRs in neuroinflammation, that results in a reduced activation of astrocytes and microglia (39). Here we reported an increased microgliosis, measured as the number of IBA1+ cells and the area covered by IBA1 ir, in the cortex and in the Hip of both 5 and 7 mo 5XFAD mice without any significant influence of NDP treatment (**Figure 5**). Analogously, in 3xTg mice a significant microgliosis was observed exclusively at 14 months of age without any effect of NDP treatment in all analyzed areas; at 9 mo, even if we did not detect any significant increase either in the number or in the area coved by microglial cells, the NDP treatment reduces microgliosis in the CA3 field of Hip of 3xTg mice (**Figure 6**). The results in 3xTg mice suggest that NDP treatment, possibly by rescuing synaptic plasticity (34), reduces microglial activation and cell proliferation that represent a hallmark of inflammation in the brain (40) to the levels of Wt. The differences observed in the two AD models used in this study, apparently contradictory, could reflect their intrinsic differences; specifically, in 5XFAD mice the process of microgliosis represents a precocious event consequent to first amyloid plaque deposition and precedes behavioral deficits (11) whereas in 3xTg mice, amyloid plaque formation occurs later and gradually increases with age and follows declined cognitive functions (23). Since the process of amyloidosis and, as a consequence, microgliosis is slower in 3xTg mice, we hypothesize that NDP treatment is more effective on microgliosis in earlier stages of AD pathology progression. Our results tally well with previous studies showing that induced MC4R activation on microglia and astrocytes elicits anti-inflammatory effects by stimulating the cholinergic pathway and the adenosine monophosphate-activated protein kinase (AMPK)/c-Jun N-terminal kinase (JNK)/p38 MAPK (41, 42); these studies demonstrated a prominent role of MCRs in reducing release of cytokines/mediators, such as TNFα, IL1ß, IL6 and reactive oxygen species, and pro apoptotic marker Bax, in experimental cerebral ischemia/reperfusion, traumatic brain injury and LPS-induced neuroinflammation (22, 42, 43). Of note, in our experimental conditions, we did not detect any global change in MC4R expression due to AD or NDP treatment suggesting a pleiotropic effect mediated by the activation of different MCRs (**Supplementary Figure 2** and **Supplementary Table 2**).

Furthermore, we only tested the expression of IBA1, a widely-used marker for microglial cells, that does not give any information about the functional/transcription state of these cells; even if we did not detect any changes in cell number and the area occupied by these cells, we cannot exclude that NDP treatment could improve microglial functions. In fact, our WB results demonstrated that the increased activation of p38 MAPK occurring in AD mice is reverted by NDP treatment in both 7 mo 5XFAD and 14 mo 3xTg mice (**Figures 7A, B**) confirming its possible anti-inflammatory role.

Finally, NDP stimulation did not cause a significant extensive reduction in GFAP ir, considered to represent an index of astrogliosis, with the exception of SUB in 9 mo 3xTg mice (**Figure 4**). Of note, also this result could be explained by specific features of amyloidosis development. In fact, the SUB of 9 mo 3xTg mice represents an instance of initial eAß deposition in the presence of iAß accumulation. Other regions of 3xTg mice show only iAß accumulation whereas SUB in 14 mo 3xTg mice (similarly to 5 or 7 mo 5XFAD mice) have extensive deposition of eAß. Therefore, we speculate that NDP treatment could exert some protective effect on astrocytes only at specific time window/stage of AD progression, precisely, at early stage of eAß deposition. In addition, since GFAP ir does not label all astrocytic populations (44, 45), our data do not exclude that NDP treatment may have a more pervasive protective effect on other astrocytic populations. Indeed, it is known that GFAP+ astrocytes contain subpopulations with different functional roles, such as A1 neurotoxic astrocytes. In fact, a recent study demonstrated that MCR stimulation in 7 mo APP/PS1 mice reduces neurotoxic A1 subtype of reactive astrocyte, which represents the major astrocytic subtype that mediates astrocytic toxicity in AD (40). Further studies with A1-specific markers may show alterations in astrocytic subpopulations that have been overlooked in present analysis.

This study has some intrinsic limitations. First, we focused on the effect of NDP on AD progression using two animal models at two ages, and to limit the number of mice in the behavioral experiments, we did not include Wt groups with NDP treatment, since we previously demonstrated the absence of any NDP effects on learning and memory in Wt mice (10, 21). However, we cannot exclude that NDP treatment induces some histological or biochemical effects in Wt mice.

Second, our *ex vivo* analysis conducted in whole hippocampi does not allow to unveil NDP effects on target proteins in specific cell populations. For example, even if globally we were able to detect few changes in some protein expression (see **Supplementary Table 2**), we cannot exclude that changes occur in restricted cell populations especially in close proximity to AD-like neuropathology. Further studies are needed to study those effects on neuronal and glial population.

Finally, even if this study was conducted in multiple animal models of AD and behavioral results were reproducible among them, these models only mimic some aspects of the typical late-onset AD dementia in humans and, for this reason, the translational predictability of present results into clinic is not immediate.

To conclude, here we investigated and compared, for the first time, the effect of NDP treatment in two animal models of AD, 5XFAD and 3xTg mice, at different phases of AD-like pathology progression. Our results clearly demonstrated that, even if systemic NDP treatment exerted only minor effects on Aβ burden and neuroinflammation, cognitive improvements are evident in both mouse models and at different stages of AD progression.

## Data availability statement

The original contributions presented in the study are included in the article/**Supplementary Material**. Further inquiries can be directed to the corresponding author.

## Ethics statement

The animal study was reviewed and approved by Committee on Animal Health and Care of the University of Modena and Reggio Emilia and Italian Minister of health.

## Author contributions

ED, EV, WL, MB, AV: mouse breeding, *in vivo* investigation, data collection; AO: biochemical analysis; AV and DG: data collection and curation, formal analysis, study supervision and validation; AV: original draft preparation; MZ, ED, EV, DG, CB: writing - review & editing; DG: Conceptualization and funding acquisition. All authors have read and agreed to the published version of the manuscript.
