# Supplementary material for: Melanocortin receptor agonist NDP-α-MSH improves cognitive deficits and microgliosis but not amyloidosis in advanced stages of AD progression in 5XFAD and 3xTg mice
Source: Front Immunol. 2023 Jan 10;13:1082036. doi: 10.3389/fimmu.2022.1082036 (PMC9871936; doi:10.3389/fimmu.2022.1082036)
Supplement: Supplementary file 1 [file DataSheet_1.docx]

|  |  |  | **5XFAD 5 mo** | **5XFAD 7 mo** | **3xTg 9 mo** | **3xTg 14 mo** |
| --- | --- | --- | --- | --- | --- | --- |
| **Fig. 2-3: 6E10** | % Area | CTX | F(1,22)=1.524, p=0.231 | F(1,12)=0.291, p=0.601 | F(1,12)=0.339, p=0.541 | F(1,14)=0.045, p=0.835 |
|  |  | DG | F(1,19)=0.013, p=0.959 | F(1,12)=0.641, p=0.440 | nd | nd |
|  |  | CA3 | F(1,21)=4.854, p=0.039 * | F(1,12)=2.654, p=0.132 | F(1,11)=0.098, p=0.760 | F(1,15)=1.074, p=0.318 |
|  |  | CA1 | F(1,21)=0.953, p=0.341 | F(1,12)=1.386, p=0.264 | F(1,11)=0.102, p=0.756 | F(1,15)=0.009, p=0.925 |
|  |  | SUB | nd | nd | F(1,12)=1.067, p=0.324 | F(1,14)=2.932, p=0.111 |
|  | Count | CTX | F(1,22)=0.253, p=0.620 | F(1,12)=1.743, p=0.214 | F(1,12)=1.012, p=0.336 | F(1,14)=0.009, p=0.927 |
|  |  | DG | F(1,22)=0.114, p=0.739 | F(1,12)=0.204, p=0.660 | nd | nd |
|  |  | CA3 | F(1,21)=0.22, p=0.883 | F(1,12)=0.113, p=0.743 | F(1,11)=0.099, p=0.761 | F(1,15)=0.518, p=0.483 |
|  |  | CA1 | F(1,21)=1.569, p=0.225 | F(1,12)=4.171, p=0.066 | F(1,11)=0.087, p=0.774 | F(1,15)=3.300, p=0.091 |
|  |  | SUB | nd | nd | F(1,13)=0.493, p=0.496 | F(1,14)=0.010, p=0.920 |
| **Fig. 4: GFAP** | % Area | DG | F(2,29)=0.633, p=0.539 | F(2,20)=14.291, p<0.001 *** | F(2,16)=9.835, p=0.002 ** | F(2,20)=4.569, p=0.025 * |
|  |  | CA3 | F(2,29)=2.285, p=0.121 | F(2,20)=5.460, p=0.014 * | F(2,17)=7.205, p=0.006 ** | F(2,20)=3.044, p=0.073 |
|  |  | CA1 | F(2,29)=2.071, p=0.146 | F(2,20)=51.650, p<0.001 *** | F(2,17)=6.301, p=0.010 * | F(2,20)=2.967, p=0.077 |
|  |  | SUB | nd | nd | F(2,17)=7.554, p=0.005 ** | F(2,20)=8.680, p=0.002 ** |
| **Fig. 5-6: IBA1** | % Area | CTX | F(2,29)=13.796, p<0.001 *** | F(2,16)=8.446, p=0.004 ** | F(2,16)=0.400, p=0.678 | F(2,22)=3.259, p=0.060 |
|  |  | DG | F(2,29)=3.910, p=0.032 * | F(2,17)=7.746, p=0.005 ** | F(2,15)=1.252, p=0.318 | F(2,22)=7.043, p=0.005 ** |
|  |  | CA3 | F(2,29)=0.946, p=0.401 | F(2,17)=2.879, p=0.087 | F(2,16)=4.263, p=0.036 * | F(2,22)=4.926, p=0.018 * |
|  |  | CA1 | F(2,29)=2.034, p=0.150 | F(2,17)=4.756, p=0.025 * | F(2,16)=2.635, p=0.107 | F(2,22)=5.050, p=0.017 * |
|  |  | SUB | nd | nd | F(2,17)=1.327, p=0.295 | F(2,22)=13.444, p<0.001 *** |
|  | Count | CTX | F(2,29)=15.175, p<0.001 *** | F(2,16)=18.781, p<0.001 *** | F(2,16)=2.2446, p=0.123 | F(2,22)=4.769, p=0.020 * |
|  |  | DG | F(2,29)=7.184, p=0.003 ** | F(2,17)=24.065, p<0.001 *** | F(2,15)=1.894, p=0.190 | F(2,22)=0.425, p=0.657 |
|  |  | CA3 | F(2,29)=8.867, p=0.001 ** | F(2,17)=6.256, p=0.011 * | F(2,16)=3.254, p=0.069 | F(2,22)=2.701, p=0.092 |
|  |  | CA1 | F(2,29)=5.526, p=0.010 * | F(2,17)=3.736, p=0.048 * | F(2,16)=10.484, p=0.002 ** | F(2,22)=7.709, p=0.003 ** |
|  |  | SUB | nd | nd | F(2,16)=3.254, p=0.069 | F(2,22)=14.169, p<0.001 *** |
| **Fig. 7: WB** | OD | pAPP T668 | nd | F(2,41)=15.312, p<0.001 *** | nd | F(2,11)=26.27, p<0.0001 **** |
|  |  | pTau S202 | nd | F(2,27)=14.593, p<0.001 *** | nd | F(2,11)=27.31, p<0.0001 **** |
|  |  | pTau T181 | nd | F(2,27)=21.115, p<0.001 *** | nd | F(2,11)=22.11, p=0.0001 *** |
|  |  | p38 MAPK | nd | F(2,25)=3.448, p=0.049 * | nd | F(2,11)=9.661, p=0.0038 ** |

**Supplementary Table 1:** statistical reports of one-way ANOVA for all IHC and WB analysed parameters; *p<0.05, **p<0.01, ***p<0.001, ****p<0.0001.

Abbreviations: nd: not determined; CTX = cortex; DG = dentate gyrus; CA3 = cornu Ammonis 3; CA1 = cornu Ammonis 1; SUB = subiculum; OD = optical density.

|  |  | **ANOVA results** | **Bonferroni multiple comparison** |
| --- | --- | --- | --- |
| **5XFAD** | pErk | F(2,27)=0.946, p=0.402 | ns |
|  | TNF-α | F(2,25)=1.015, p=0.378 | ns |
|  | pTau S396 | F(2,25)=4.395, p=0.024 * | 5XFAD sal vs 5XFAD NDP p=0.030 * |
|  | pJNK | F(2,27)=1.323; p=0.284 | ns |
|  | Aβ1-42 | F(2,25)=49.289, p<0.001 *** | B6SJL sal vs 5XFAD p<0.001 *** |
| **3xTg** | pErk | F(2,11)=3.739, p=0.058 | C57BL/6J vs 3xTg sal p = 0.058 |
|  | TNF-α | F(2,11)=0.609, p=0.5614 | ns |
|  | MCR-4 | F(2,11)=0.7594; p=0.491 | ns |
|  | BACE1 | F(2,11)=23.57; p=0.0001 *** | C57BL/6J vs 3xTg sal p=0.0001 ****; vs 3xTg NDP p=0.0012 ** |

**Supplementary Table 2:** data related to protein expression are shown as mean OD ± SEM and were analyzed according to one-way ANOVA followed by Bonferroni correction. *p<0.05, **p<0.01, ***p<0.001, ****p<0.0001.


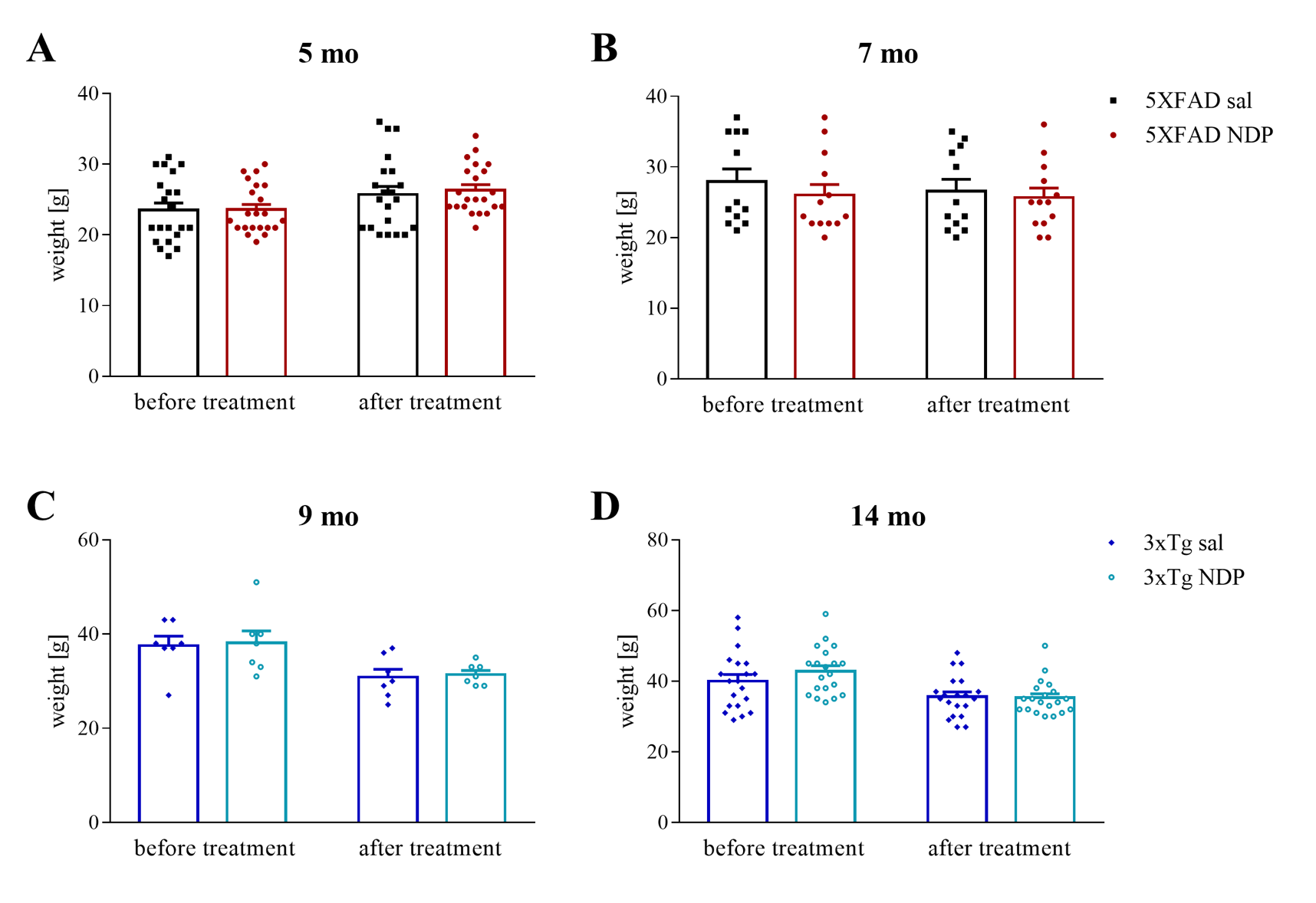


**Supplementary Figure 1: Body weight of 5XFAD (A-B) and 3xTg (C-D) mice is not influenced by NDP treatment.** Data are shown as mean ± SEM and were analyzed according to one-way repeated measures ANOVA. Experimental groups: 5XFAD sal: 5 mo: n=21; 7 mo: n=12; 5XFAD NDP: 5 mo: n=21; 7 mo: n=13; 3xTg sal: 9 mo: n=7; 14 mo: n=20; 3xTg NDP: 9 mo: n=7; 14 mo: n=20.

**
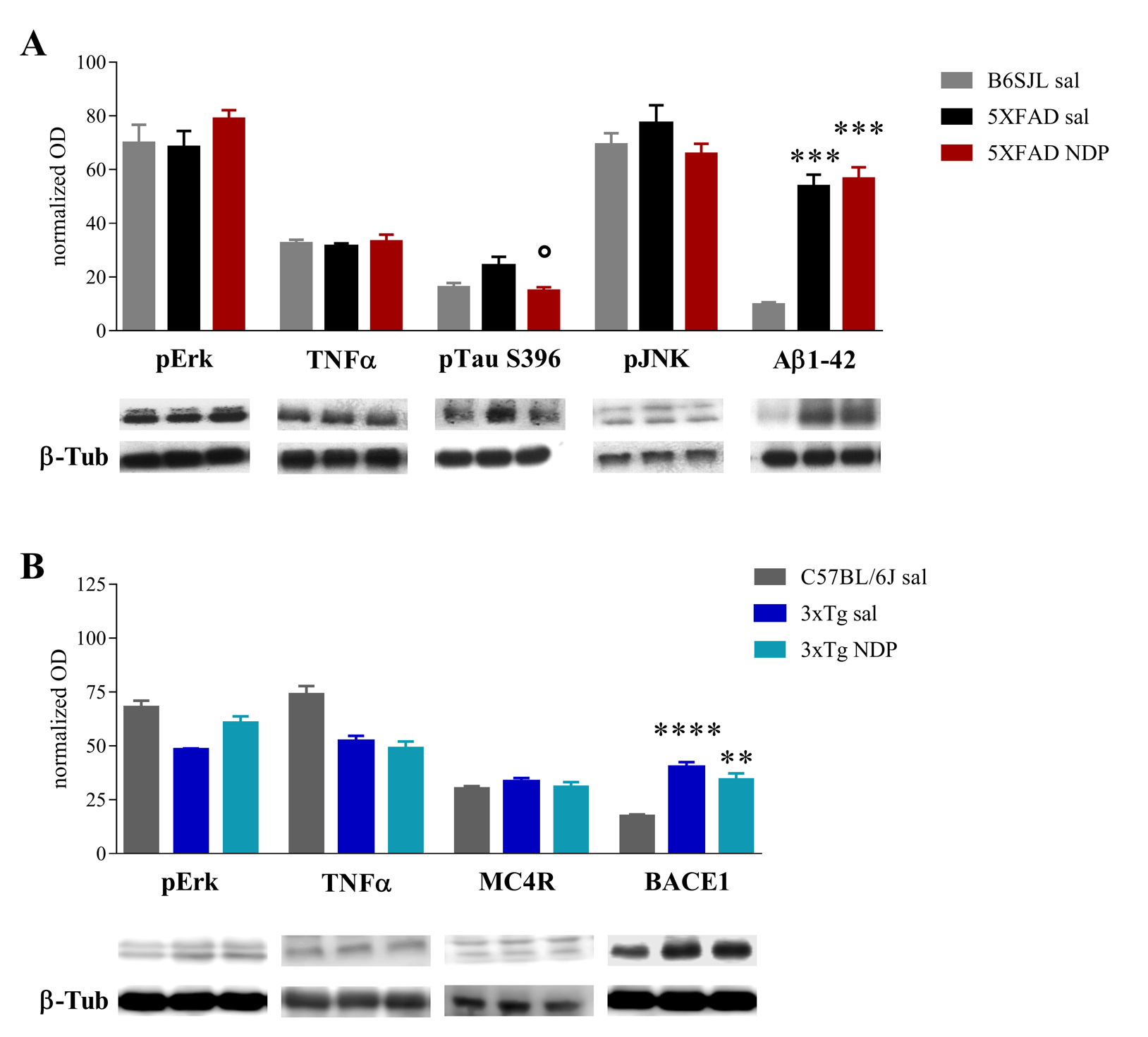
**

**Supplementary Figure 2: Effects of NDP treatment on specific markers in the hippocampus of 7 mo 5XFAD and 14 mo 3xTg mice.** A) Protein levels measured by western blot analysis in B6SJL (light grey bar) and 5XFAD mice treated with saline (black bar) or NDP (red bar) and B) in C57BL/6J (grey bar) and 3xTg mice treated with saline (blue bar) or NDP (grey bar). Ab-specific optical density (OD) was normalized over ß-Tub signal. Data are shown as mean ± SEM and were analyzed according to one-way ANOVA followed by Bonferroni correction (* vs Wt; ° vs Tg sal °p<0.05, **p<0.01, ***p<0.001, ****p<0.0001).

Experimental groups: B6SJL sal: 7 mo=8-10; 5XFAD sal: 7 mo: n=8-10; 5XFAD NDP: 7 mo: n=10; C57BL/6J sal: n=4; 3xTg 14 mo: n=5; 3xTg NDP: 14 mo: n=5.
